# Evaluation of a Text Messaging Intervention to Promote Preconception Micronutrient Supplement Use: Feasibility Study Nested in the Healthy Life Trajectories Initiative Study in South Africa

**DOI:** 10.2196/37309

**Published:** 2022-08-18

**Authors:** Larske M Soepnel, Michelle C McKinley, Sonja Klingberg, Catherine E Draper, Alessandra Prioreschi, Shane A Norris, Lisa J Ware

**Affiliations:** 1 SAMRC/Wits Developmental Pathways for Health Research Unit Faculty of Health Sciences University of the Witwatersrand Johannesburg South Africa; 2 Julius Global Health, Julius Center for Health Sciences and Primary Care University Medical Center Utrecht Utrecht University Utrecht Netherlands; 3 DSI-NRF Centre of Excellence in Human Development University of the Witwatersrand Johannesburg South Africa; 4 Centre for Public Health School of Medicine, Dentistry, and Biomedical Sciences Queen's University Belfast Belfast United Kingdom; 5 School of Health and Human Development University of Southampton Southampton United Kingdom

**Keywords:** preconception health, micronutrient supplements, adherence, behavioral, SMS text messaging intervention, mobile health, mHealth, radio serial, mobile phone

## Abstract

**Background:**

Social messaging strategies such as SMS text messaging and radio are promising avenues for health promotion and behavior change in low- to middle-income settings. However, evidence of their acceptability, feasibility, and impact in the context of young women’s health and micronutrient deficiencies is lacking.

**Objective:**

This study aimed to evaluate the feasibility of an automated 2-way text messaging intervention nested in an ongoing preconception health trial, the Healthy Life Trajectories Initiative (HeLTI; HeLTI *Bukhali*) in Soweto, South Africa. Second, we aimed to evaluate the acceptability of a health promotion radio serial, which aired concurrently in the region.

**Methods:**

In this feasibility study, 120 participants enrolled in HeLTI *Bukhali* between November 2020 and February 2021 received the 6-month 2-way text messaging intervention. Quantitative and qualitative data on intervention acceptability, usability, interaction, perceived benefit, and fidelity were collected during 5 focus group discussions (FGDs) and from study data logs. During the FGDs, data were collected on the acceptability of the radio serial. Following the text messaging intervention, capillary hemoglobin levels were assessed, and a participant questionnaire provided information on adherence and attitudes toward supplements. The text messaging control group comprised the first 120 women recruited from November 2019 to February 2020, who received the *Bukhali* intervention but not the text messages. Statistical significance testing and a linear mixed model were used for indicative effect comparisons between the text message–receiving and control groups.

**Results:**

The text messaging intervention was found to be acceptable and to have perceived benefits, including being reminded to take supplements, gaining knowledge, and feeling supported by the study team. The use of the 2-way text messaging reply function was limited, with only a 10.8% (13/120) response rate by week 24. Barriers to replying included a lack of interest or phone credit and technical issues. Regarding the indicative effect, participants receiving the text messages had higher self-reported adherence at follow-up than the text messaging control group (42/63, 67% vs 33/85, 39% taking supplements every time; *P*=.02), and altitude-adjusted hemoglobin increased more between baseline and follow-up in the SMS text message–receiving group than in the text messaging control group (1.03, 95% CI 0.49-1.57; *P*<.001). The radio serial content was acceptable, although few participants reported exposure before the FGD.

**Conclusions:**

Women reported that the text messaging intervention was useful and described the benefits of receiving the messages. Examination of hemoglobin status indicated a promising beneficial effect of text messaging support on adherence to micronutrient supplementation, requiring further exploration through randomized controlled studies. Health promotion through radio and text messages were both found to be acceptable, although more research into the radio serial reach among young women is needed.

**Trial Registration:**

Pan African Clinical Trials Registry (PACTR) PACTR201903750173871; https://tinyurl.com/4x6n32ff

## Introduction

### Background

Women’s health and nutritional status before and during pregnancy are increasingly being identified as important determinants of their future health, pregnancy success, and the health of the next generation [1]. However, across high-, middle-, and low-income countries, both over- and undernutrition remain prevalent before and during pregnancy, and nonadherence to nutritional recommendations is a global issue [1-7]. Micronutrient deficiencies persist in South Africa, with anemia affecting 23% to 31% of women of reproductive age in urban settings [8,9]. In this context, preconception health and its consequences have also been identified as important knowledge gaps among young women [10].

Using digital spaces for health promotion and health behavior change is an increasing global phenomenon. In South Africa, radio is the most consumed form of media, with an estimated 37.8 million weekly listeners, of whom approximately 30% listen via mobile phones [11]. Although radio messaging has shown promise for improving health knowledge [12], health behavior changes may be more effectively achieved when messaging is targeted to specific population subgroups and tailored to the needs of the individual [13].

SMS text messaging interventions are a potential avenue for providing targeted and tailored behavior change support, reinforced by the high level of mobile phone penetration in many low- to middle-income settings. For example, approximately 96% of households in South Africa in 2018 had a mobile phone, and of the country’s population, 82% were estimated to have a smartphone subscription (smartphone penetration) [14]. Increasing data support the use of various SMS text messaging interventions to improve appointment attendance, medication adherence, and risk-related behavior change, mostly in the context of chronic diseases [15-19]. However, systematic reviews from low- to middle-income countries have found mixed or inconclusive evidence of the impact of SMS text messaging interventions, which are often not explicitly designed using behavior change theory [20-22]. In South Africa, SMS text messaging interventions have shown highly promising results for HIV care adherence, support, and education [23-25], as well as for pregnant and postpartum women, through the National MomConnect SMS text message health messaging program [26,27]. Data from high-income settings suggest that a 2-way SMS text messaging intervention can support nutritional behavior change, resulting in weight loss and maintenance in the postpartum period [28], and that a mobile app can effectively improve preconception nutrition [29]. However, to the best of our knowledge, the use of SMS text messaging to improve nutritional status during the preconception period has not been previously explored.

### Objectives

The main aim of this study was to evaluate the feasibility of a tailored 6-month SMS text messaging intervention to support adherence to preconception micronutrient supplementation in women enrolled in the Healthy Life Trajectories Initiative (HeLTI) *Bukhali* trial, a complex preconception health trial in Soweto, South Africa. This included an evaluation of the acceptability, usability, perceived benefit, fidelity, cost, and indicative effects of the SMS text messaging intervention for promoting supplement adherence. The second objective was to evaluate the acceptability of a radio serial and accompanying Facebook page aimed at supporting health promotion in the trial setting, which was aired toward the end of the SMS text messaging intervention.

## Methods

### Setting and Population

This study was nested in the intervention group of the South African site of the HeLTI *Bukhali* study, a randomized controlled trial that aimed to evaluate a multifaceted intervention for promoting the health of women of reproductive age (18-28 years) [30,31]. Similar trials are ongoing in Canada, India, and China in collaboration with the World Health Organization. HeLTI *Bukhali* is based in Soweto, a historically disadvantaged township bordering Johannesburg, with approximately 1.3 million residents, and recruitment started in 2018. The intervention was delivered by research staff trained in healthy conversation skills [32], who distributed educational resource material and provide health feedback in terms of BMI, hemoglobin, blood pressure, hemoglobin A_1c_ to assess hyperglycemia, HIV testing, and mental health and facilitated sessions to support improved health behaviors around nutrition, physical activity, sleep, health monitoring (eg, HIV testing), and other goals identified by the participants. An aspect of the HeLTI *Bukhali* intervention is the provision of multimicronutrient supplements based on participants’ anemia status. Women receive a supplement containing, among other micronutrients, 27 mg iron twice per week if they are nonanemic (capillary hemoglobin ≥12 g/dL) and daily if they are mildly anemic (hemoglobin <12 g/dL), with women who are severely anemic (hemoglobin <7 g/dL) receiving referrals to receive the current standard of care, comprising further assessment, supplementation, and additional management as required. The trial’s control group was contacted once a month through telephone, SMS text message, or email to deliver information on *life skills* not directly related to health and had access to standard health services such as HIV and pregnancy testing. The intervention and control arms of the trial were included in the trial program for a total of 18 months and were followed throughout pregnancy and postpartum periods in case of pregnancy within the 18-month time frame [31].

The exclusion criteria for HeLTI *Bukhali* were a diagnosis of type 1 diabetes, cancer, or epilepsy; the presence of an intellectual disability that hinders informed consent; and being unwilling or unable to consent. For this feasibility study, only women recruited into the preconception intervention group of the trial were included. This study evaluated 2 distinct remote media approaches (SMS text messaging and radio combined with Facebook) in the same trial.

**Figure 1:**
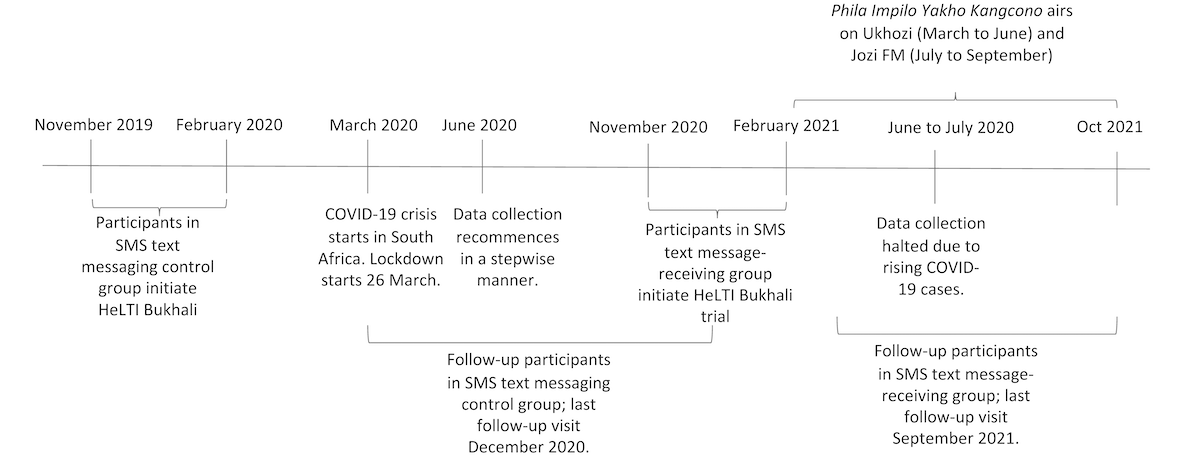
Timeline of SMS text messaging feasibility study and airing of the radio serial in the context of the COVID-19 pandemic in South Africa. HeLTI: Healthy Life Trajectory Initiative.

As indicated in the timeline in Figure 1, the first 120 women enrolled in HeLTI *Bukhali* between November 1, 2020, and February 2021, who consented to the SMS text messaging substudy, received the SMS text messaging intervention. This *SMS text message–receiving* group was asked to participate in this study’s focus group discussions (FGDs), in which the acceptability of the health promotion radio serial was also evaluated.

In addition, for the preliminary evaluation of the indicative effect of the SMS text messaging intervention, a comparison group receiving the *Bukhali* intervention but not receiving the SMS text messages (*SMS text messaging control group* from here on) comprised the first 120 women who were recruited to the HeLTI *Bukhali* intervention arm from November 2019 onward until the target sample size was attained without any matching the SMS text message–receiving group. The time frame of the SMS text messaging control group recruitment was chosen to avoid seasonal variability in micronutrient status. This also avoided the major hiatus in data collection in response to the COVID-19 crisis that started in March 2020. Follow-ups for participants in the SMS text message–receiving and SMS text messaging control groups were conducted between May and October 2021 and May and December 2020, respectively. The delays in follow-up in both groups were largely attributable to the COVID-19 pandemic.

### The HeLTI Bukhali Text Messaging Intervention

The HeLTI *Bukhali* SMS text messaging intervention and message bank were developed based on existing evidence and the behavior change theory. Textbox 1 provides an overview of the SMS intervention according to the TIDieR (Template for Intervention Description and Replication) checklist. The Health Actions Process Approach (HAPA) [33] was the main guiding theoretical approach for developing the SMS text message library. The HAPA is a sociocognitive model encompassing 2 phases: the motivation phase, including outcome and risk perception and self-efficacy, and the volitional phase, including action-oriented processes such as action planning and maintenance of self-efficacy. HAPA has previously been shown to successfully inform medication adherence and UK-based SMS text messaging interventions [28,34,35]. The message bank was developed with input from young women in Soweto, including lessons from formative work for HeLTI *Bukhali* around barriers to supplement use [31], and was tested for any content or technical issues before this feasibility study.

**Reporting of the Template for Intervention Description and Replication checklist items for the Healthy Life Trajectories Initiative (HeLTI) Bukhali SMS text messaging intervention.**

**Template for Intervention Description and Replication checklist item and brief description**

- Brief name
  The brief name is HeLTI Bukhali SMS text messaging intervention.
- Why
  A lack of adherence to micronutrient supplementation is a prevalent barrier to improving micronutrient deficiencies in the preconception period. SMS text messaging interventions are accessible and have shown potential as a cost-effective avenue to support behavior change in middle- and low-income settings. Therefore, this SMS text messaging intervention aims to improve adherence to and knowledge around micronutrient supplements in women of reproductive age. The intervention development is grounded in behavior change theory, specifically the Health Action Process Approach.
- What (materials)
  The intervention consisted of comprised 48 SMS text messages delivered over the course of 24 weeks (2 per week). Messages were sent around midday on varying days of the week, as programmed from the day of initial HeLTI Bukhali enrollment. In addition to an introductory message, the 3 main types of SMS text messages were as follows: Health literacy messages: weekly educational messages on the contents and potential benefits of the micronutrient supplement, how they work, potential side effects, the importance of a balanced diet, tips on when to take the supplements, and how to remember them more easily Adherence messages: weekly 2-way messages querying whether participants had taken their supplements in the week (“did you take all your micronutrient pills this week?”); participants could reply “yes” or “no,” prompting a second message asking the main reason for not taking the supplement if the answer was “no” (“I forgot, I ran out of pills, the pills made me feel unwell, other”) Side effect–reporting messages: 2-way messages sent in weeks 2, 8, 13, and 20 instead of the health literacy message, asking participants if they had experienced any side effects from the supplements, to which participants could reply “yes” or “no,” and if they replied “yes,” a second message was sent asking which symptoms were present.
  The message bank can be accessed from the corresponding author upon reasonable request. Participants were automatically supplied with ZAR 5 prepaid airtime weekly, which sufficiently covered the cost of responding to an SMS text message through any available provider.
- What (procedure)
  SMS text messages were automatically sent twice a week to participant phone numbers, using Twilio Inc, and airtime was supplied automatically using FlickSwitch Control South Africa (Cape Town). The intervention evaluation was nested in the HeLTI South Africa Bukhali trial, which evaluates a complex intervention (education, social support, behavior change, and micronutrient supplement) [30,31]. Both the SMS text message–receiving and SMS text messaging control groups received the intervention arm of HeLTI Bukhali, although the control arm did not receive the SMS text messaging intervention in any form. As part of the trial, multimicronutrient supplements are provided based on the participants’ anemia status (see Setting and Population section for more details).
- Who provided
  The SMS text messaging intervention was developed by the research team with input from young women in Soweto during the development phase. The delivery of the SMS text messaging intervention was automated.
- How
  The mode of delivery was through SMS text messages. In the control group, no SMS text messages were delivered.
- Where
  The intervention was delivered through SMS text messages. Participants were based in Soweto, a historically disadvantaged township bordering Johannesburg, with approximately 1.3 million residents.
- When and how much
  The SMS text messaging intervention was delivered to 120 participants newly enrolled in HeLTI Bukhali between November 1, 2020, and February 2021 who additionally consented to the SMS text messaging substudy. The SMS text messaging intervention was delivered twice weekly for 6 months at midday on varying days of the week, as automatically scheduled based on the day of the participants’ initial enrollment in HeLTI Bukhali. For the feasibility study, the last participant received the last message in August 2021. The SMS text messaging control group comprised 120 HeLTI Bukhali participants who enrolled between November 2019 and February 2020, receiving the Bukhali intervention but no SMS text messages.
- Tailoring
  The SMS text messaging intervention was not tailored to individual participants.
- Modification
  The SMS text messaging intervention was not modified during the course of the feasibility study.
- How well (planned)
  The fidelity of the planned intervention in terms of messages received, airtime received, and presence of any technical errors will be reviewed using a log of the automated SMS text messaging and airtime delivery systems.
- How well (actual)
  See the Results section.

As described in Textbox 1, participants received 2 messages every week for 24 weeks, with messages comprising health literacy, adherence, and side effect reporting. From the time participants were recruited to HeLTI *Bukhali*, SMS text messages were sent and received using an automated SMS text message delivery platform—Twilio Inc. To avoid nonparticipation in the 2-way SMS text messages because of a lack of funds, participants were automatically supplied with 5 South African Rand (ZAR 5, around US $0.30) prepaid minutes of phone credit (“airtime”) weekly, which sufficiently covered the cost of responding to an SMS text message through any available provider, using FlickSwitch SIMControl South Africa (Cape Town). The successful delivery of the SMS text messages and airtime could be monitored through these respective platforms and was evaluated through qualitative data collection.

### The Phila Impilo Yakho Kangcono Radio Serial

The *Phila Impilo Yakho Kangcono* (translating to “Live Your Best Life”) radio serial was designed as media support for the HeLTI trial, and an overview based on the TIDieR checklist is provided in Textbox 2. The weekly radio serial targeted young people and aimed to promote well-being and health. It was aired in isiZulu and English between March and June 2021 on a Sunday youth show on Ukhozi FM, South Africa’s largest radio station, and on Jozi FM, a local radio station popular in Soweto, from July to September 2021. An associated Facebook page was created and referred to in the radio episodes [36]. The content of each episode was designed by an independent production team working with and guided by young adults (both men and women) from Soweto and the research team.

**Reporting of Template for Intervention Description and Replication checklist items for the Phila Impilo Yakho Kangcono radio serial intervention.**

**Template for Intervention Description and Replication checklist item and brief description**

- Brief name
  The brief name is the Phila Impilo Yakho Kangcono radio serial.
- Why
  Preconception health and its significance are important knowledge gaps among young women, and radio is one of the most consumed forms of media in South Africa. Therefore, population-based health promotion through radio could be an accessible way of increasing health awareness and well-being among young people in South Africa.
- What (materials)
  The intervention comprised 11 radio serial episodes of 15 to 20 minutes in English and isiZulu, designed to cover health and well-being topics identified as important by youth, such as mental health (eg, suicide and depression), diabetes, gender-based violence, hypertension, HIV, healthy food, and community gardens. In addition, an associated Facebook page was created to promote the radio serial and for listeners to comment and catch up on missed episodes. Each of the radio serial episodes can be accessed on the Facebook page, which is fully accessible to the public [36].
- What (procedure)
  The radio serial was aired weekly on 2 radio stations—Ukhozi FM (South Africa’s largest radio station with a listener base of around 8 million people) and Jozi FM (a local radio station popular in Soweto)—with referral to the associated Facebook page.
- Who provided
  The intervention was delivered over the radio and therefore not provided by individual care providers.
- How
  The mode of delivery was radio (and Facebook), designed as a media campaign to be delivered on a population level rather than to individual participants.
- Where
  The intervention was delivered through radio on Ukhozi FM and Jozi FM and through the associated Facebook page.
- When and how much
  The radio serial aired once a week at 2:30 PM on Sundays from March to June 2021 on Ukhozi FM and at 10:30 AM from July to September 2021 on Jozi FM.
- Tailoring
  The radio serial was not personalized or tailored.
- Modification
  The intervention was not modified over the course of the study.
- How well (planned)
  Not applicable.
- How well (actual)
  Not applicable.

### Ethics Approval

The human research ethics committee (Medical) at the University of Witwatersrand approved this study (M171137 and M1811111). Participants gave written informed consent before enrollment into the study and provided additional written informed consent before participating in the recorded FGDs.

### Outcomes

This mixed methods study adopted a parallel-convergent approach, using a combination of quantitative and qualitative data sources to address feasibility outcomes [37]. The framework for evaluating young women’s perceptions of the HeLTI *Bukhali* SMS text messaging intervention and the radio serial and associated Facebook pages are outlined in Table 1 and are based on the existing literature on process evaluation [38,39]. The main outcomes of interest for evaluating women’s perceptions of the SMS text messaging intervention were acceptability, usability and level of interaction, perceived benefit, intervention cost and fidelity, and indicative effects. For the *Phila Impilo Yakho Kangcono* radio serial and associated Facebook page, the main outcome was the acceptability of the radio serial and the associated Facebook page.

**Table 1:** Main outcomes and data sources for the study.

Objective			Main question addressed		Data source
**Objective 1: feasibility of** **Healthy Life Trajectories Initiative** *Bukhali* **SMS** **text messaging** **intervention**					
	Acceptability	To what extent was the intervention delivery agreeable and acceptable to participants?		Qualitative data from FGDs^a^ with participants	
	Usability and interaction	To what extent could the intervention be used and was the intervention used adequately by the participants?		Data log of the number of participants using the 2-way SMS text messaging system throughout the 6-month intervention; qualitative data from FGDs with participants	
	Perceived benefit	What were the perceived benefits of the intervention? Were there any unintended consequences of the intervention?		Qualitative data from FGDs with participants	
	Fidelity of intervention delivery	To what extent was the intervention delivered as designed?		Log of messages received, airtime received, and technical errors	
	Cost of intervention delivery	What costs were associated with intervention delivery?		Log of cost of intervention	
	Indicative effect	What are the indicative effects of the intervention on self-reported adherence, attitudes toward micronutrient supplements, and hemoglobin level at follow-up?		Quantitative baseline and follow-up hemoglobin values; quantitative surveys of attitudes to micronutrient supplements at follow-up	
**Objective 2: acceptability of the Phila Impilo Yakho Kangcono radio serial**					
	Acceptability	To what extent was the intervention delivery agreeable and acceptable to participants? To what extent had participants been exposed to the radio serial?		Qualitative data from FGDs with participants	

^a^FGD: focus group discussion.

### Data Collection

#### HeLTI Bukhali Text Messaging Intervention

Qualitative data on the acceptability, usability, interaction with, and perceived benefit of the SMS text messaging intervention were collected during 5 FGDs, including 2 to 9 participants. These were conducted by trained research staff from Soweto using a topic guide developed by the research team and were organized and attended by LMS. Participants were invited at random from those receiving SMS text message notifications 4 to 8 months after the initiation of the study. Refusals to participate in the FGDs were because of having moved away from Soweto, having other commitments, or being ill on the day of the FGD. Refreshments were provided at the FGD, which took 1 to 2 hours. The FGDs were conducted in English and in the participants’ preferred language and were audio recorded (Philips Digital Voice Recorder DVT4110 and DVT1150). The recordings were transcribed verbatim but were anonymized and translated into English where necessary.

To evaluate the indicative effect of SMS text messaging intervention, data were captured and managed using REDCap (Research Electronic Data Capture; Vanderbilt University) [40]. Data were collected by trained research assistants at the research unit in Soweto. At baseline, a validated questionnaire was used to gather information on participant age, attitudes toward and confidence in supplement use, parity, country in which the participant was born, their home language, education level, employment status, and food security [41]. Participants were categorized as *at risk of food insecurity* if they answered *yes* to one of the 3 questions in the food security questionnaire (“Does your household ever run out of money to buy food?”; “Do you ever cut the size of meals or skip meals because there is not enough money to buy food?”; “Do you go to bed hungry because there is not enough money to buy food?”) and as *food insecure* if they answered *yes* to ≥2 of the questions, as previously described in our setting [42].

At a follow-up visit conducted approximately 6 months after initial recruitment for both the SMS text message–receiving and SMS text messaging control groups, a questionnaire was used to gather data on self-reported adherence to the micronutrient supplements (1=I never took them to 5=I took them every time), participants’ perceived understanding of the condition of anemia, perceived health improvement because of the supplement, fears and side effects related to the supplement, and main reasons for not taking the supplement. At both baseline and follow-up, hemoglobin levels were measured using a Hemocue 201 device. As Soweto is situated >1700 m above sea level, hemoglobin was altitude adjusted by –0.5 g/dL, according to the World Health Organization recommendation [43], as has previously been evaluated in our setting [44]. The difference in hemoglobin levels from baseline to follow-up (Δhemoglobin) was calculated (hemoglobin follow-up – hemoglobin baseline).

Data obtained from the log of the automated SMS text messaging system also provided information on participant interaction with the intervention (rate of response to 2-way SMS text messaging features) and intervention fidelity (number of messages successfully received, technical failures, and airtime received by participants). The study records provided information on the total intervention costs per participant.

#### Phila Impilo Yakho Kangcono Radio Serial

During the 5 FGDs described in detail previously, all participants played 1 to 2 episodes of the *Phila Impilo Yakho Kangcono* radio serial and were shown the associated Facebook page to ensure exposure among all participants. Subsequently, data were collected on the acceptability of the radio serial. These data were recorded and transcribed as described previously.

### Data Analysis

Qualitative data analysis was informed by the thematic analysis methods described by Braun and Clarke [45]. The analytic process and presentation of results followed the phases set out in the framework method, which is a codebook approach to thematic analysis [46]. The coding of transcripts combined a deductive approach based on the study objectives and the adapted process evaluation framework [38,39] outlined in Table 1, with inductive analysis to allow for flexibility in incorporating unforeseen findings. LMS read the transcripts to familiarize herself with the data. The initial coding of the transcripts was completed by LMS using MAXQDA software (version 20.4.1; VERBI GmbH), and preliminary themes and framework matrices were reviewed by and discussed with LJW, SK, and MM to refine the analysis.

Quantitative data analysis was performed using STATA (version 13.0, StataCorp). Baseline characteristics and SMS text message response data were described as numbers and percentages for categorical variables and mean and SD or median and IQR for continuous variables. Outcomes at the 6-month visit, including change in hemoglobin from baseline (Δhemoglobin) and change in anemia status, were compared between the participants receiving SMS text messages and the SMS text messaging control group using the Student *t* test (2-tailed) or Wilcoxon rank-sum test, depending on the normality of the data. The baseline characteristics of participants lost to follow-up in the SMS text message–receiving and SMS text messaging control groups were described and compared with followed-up participants using statistical significance testing. Mixed linear modeling was used to explore the time point–adjusted association among altitude-adjusted hemoglobin, intervention exposure, time from baseline to follow-up, and the interaction between intervention exposure and time. A second, adjusted model was run adding variables that were statistically significantly different between the SMS text message–receiving and SMS text messaging control groups at baseline.

## Results

### The HeLTI Bukhali Text Messaging Intervention

#### Participant Baseline Characteristics

**Figure 2:**
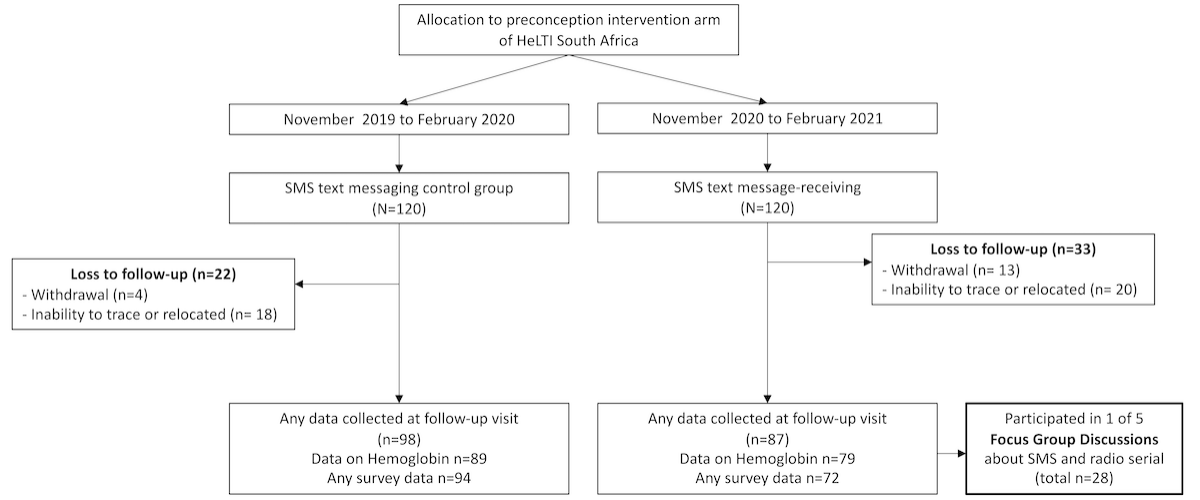
Overview of study participants during the feasibility study. HeLTI: Healthy Life Trajectory Initiative.

Figure 2 shows the participants of this study. For the SMS text messaging intervention, 72.5% (87/120) of participants in the SMS text message–receiving group and 81.7% (98/120) of participants in the SMS text messaging control group had their data collected at the follow-up visit. Of these, 90.8% (79/87) in the SMS text message–receiving group and 90.8% (89/98) in the SMS text messaging control group had hemoglobin data available from their follow-up visits. Reasons for loss to follow-up included withdrawal from HeLTI *Bukhali* (not specifically from the SMS text messaging substudy; 17/240, 7.1%; 13/120, 10.8%, in the SMS text message–receiving, and 4/120, 3.3%, in the SMS text messaging control group) and relocation or an inability to trace (38/240, 15.8%). The main baseline characteristics in the followed-up versus lost to follow-up group are provided in Table S1 in Multimedia Appendix 1, and the only statistically significant difference between these 2 groups was the number of participants born in South Africa (185/185, 100%, in the followed group vs 53/55, 96%, in the lost to follow-up group).

Table 2 shows the main baseline characteristics of the study participants. The median age was 21.5 (IQR 19-24) years, and 30.8% (74/240) of the participants had anemia (hemoglobin <11.9) at baseline. More SMS text message–receiving participants were unemployed and fewer had graduated from high school than the SMS text messaging control group participants. Moreover, the number of participants with mild or severe anemia at baseline was higher in the SMS text message–receiving group than in the SMS text messaging control group (50/120, 41.7% vs 24/120, 20%).

**Table 2:** Baseline characteristics of participants from Healthy Life Trajectories Initiative Bukhali assigned to the SMS text messaging or SMS text messaging control groups in the SMS text messaging intervention study (N=240).

Characteristics					All			Receiving SMS text messages (n=120)			SMS text messaging control (n=120)			*P* value
Age (years), median (IQR)					21.5 (19-24)			22 (20-25)			21 (19-24)			.07
Hemoglobin altitude adjusted (g/dL), median (IQR)					12.4 (11.7-13.6)			12.2 (11.5-13.2)			12.6 (12.1-13.8)			<.001^b^
**Anemia status, n (%)**														
	≥12			166 (69.2)			70 (58.3)			96 (80)			.001^b^	
	7-11.9			72 (30)			48 (40)			24 (2)			.001^b^	
	<7			2 (0.8)			2 (1.7)			0 (0)			.001^b^	
	BMI, median (IQR)			23.7 (20.8-28.1)			24.4 (21.3-29.1)			22.6 (20.2-27.8)			.05	
**Weight status, n (%)**														
	Underweight			21 (8.8)			8 (6.7)			13 (10.8)			.18	
	Normal weight			124 (51.7)			58 (48.3)			66 (55.0)			.18	
	Overweight or obese			95 (39.6)			54 (45.0)			41 (34.2)			.18	
**Attitudes toward supplements at baseline, n (%)^a^**														
	**How sure are you that you will be able to take all or most of your supplements as directed?**													
		Not at all	1 (0.6)			0 (0)			1 (1.8)			.24		
		Somewhat sure	11 (6.4)			6 (5.3)			5 (8.8)			.24		
		Very or extremely sure	159 (93)			108 (94.7)			51 (89.5)			.24		
	**How sure are you that the supplements will have a positive effect on your health?**													
		Not at all	4 (2.3)			2 (1.8)			2 (3.5)			.67		
		Somewhat sure	28 (16.4)			20 (17.5)			8 (14)			.67		
		Very or extremely sure	139 (81.3)			92 (80.7)			47 (82.5)			.67		
**Demographic characteristics, n (%)**														
	**Previous live births**													
		0	132 (55.2)			59 (49.6)			73 (60.8)			.21		
		1	76 (31.8)			42 (35.3)			34 (28.3)			.21		
		≥2	31 (13.0)			18 (15.1)			13 (10.8)			.21		
	Born in South Africa			239 (99.6)			119 (99.2)			120 (100)			.32	
	Unemployed (and not studying)			184 (76.7)			99 (82.5)			85 (70.8)			.03^b^	
	Graduated high school			145 (60.4)			64 (53.3)			81 (67.5)			.03^b^	
	**Food security^c^**													
		At risk (1)	55 (22.9)			29 (24.2)			26 (21.7)			.71		
		Food insecure (≥2)	86 (35.8)			40 (33.3)			46 (38.3)			.71		
	Current frequent smoker			30 (12.5)			15 (12.5)			15 (12.5)			.49	
	Smoked in the past year			54 (22.5)			23 (19.2)			31 (25.8)			.22	

^a^Total sample n=171; SMS text messaging group n=114; SMS text messaging control group n=57.

^b^Indicates a statistically significant difference between the SMS text message–receiving and control groups at *P*<.05 using a Mann-Whitney *U* test for continuous outcomes and chi-square statistic or Fisher exact test (if cell count <5) for categorical outcomes.

^c^Total sample n=239; SMS text messaging group n=119; SMS text messaging control group n=120.

#### Objective 1: Feasibility of the Bukhali Text Messaging Intervention

##### Acceptability

In terms of acceptability, participants liked receiving SMS text messages on their phones, which they expressed using frequently, and reported that the content was acceptable (Table 3).

The participants suggested changes that would make the SMS text messages more acceptable and useful. For example, participants suggested sending SMS text messages earlier in the morning and on days when they had to take micronutrient supplements (such as Mondays). Participants taking a daily dose of micronutrient supplements expressed the potential added value of receiving reminder messages every day. However, some participants described adherence messages as repetitive, resulting in them not always being read. Although some participants expressed that the health literacy messages were too long, others liked them and described reading them in full. Participants gave suggestions unique to their personal preferences and needs, such as receiving messages at night or preferring phone calls.

**Table 3:** Qualitative evaluation of the acceptability of the Healthy Life Trajectories Initiative Bukhali SMS text messaging intervention^a^.

Acceptability			Quotes
**Timing**			
	**In support (positive)**		
		The timing of receiving the messages was acceptable	“I think it’s fine, there’s nothing wrong with the timing...It asks you questions about the supplements and then tell you what the supplements provide you with, so I think it doesn’t cause any harm” [FGD^b^ 1] “Whatever time I get the notification I read it and see what it contains, then I’m fine, I don’t see anything wrong.” [FGD 1] “I think it’s the perfect time because they usually send an SMS in the morning.” [FGD 2]
	**Against (negative)**		
		Receiving the SMS text message at midday or later in the day was too late	“I think mornings are better because you know that when you wake up, after breakfast you take it [micronutrient supplements].” [FGD 5] “[I receive the SMS] around 12 and you find that I am in class.”
		Participants wanted to receive the message on Monday when they take their supplement	“For me the one that I want for the morning is the Monday one because when I’m from the weekend, I don’t want to lie, I forget.” [FGD 3]
		Other participants had personal timing preferences	“I prefer night time because I just take it before bed time then sleep.” [FGD 5]
	**Suggested adaptations**		
		To receive the messages early in the morning and on Mondays	“They should input it on time and not input it late, they should send it at around seven” [FGD 3] “I think they should send them Mondays and Wednesdays around 9, if you forgot, that will be a reminder that ‘I was supposed to take my supplements’” [FGD 1]
**Frequency**			
	**In support (positive)**		
		Receiving messages twice a week was acceptable	“I think it’s fine, because you have to take them twice a week. So yes, it’s OK.” [FGD 4] “it was actually a good thing getting them twice a week.” [FGD 2] “I think twice a week it’s perfect.” [FGD 2]
	**Against (negative)**		
		Receiving messages twice a week was not enough	“I think it’s too little.” [FGD 3] “I wish they would send those every day” [FGD 1]
	**Suggested adaptations**		
		To receive the messages every day, particularly for those taking their supplements daily	“At least every day, for me.” [FGD 3] “You should take them every day if your iron is low, so the SMS’s should also come every day. If your iron is OK; they should come in on your designated days to take the supplements.” [FGD 5]
		However, some expressed that receiving the same messages every day would be too repetitive	“Sometimes the SMS’s say the same thing; so do you think it’s right that we get those every day?” [FGD 5]
**Content**			
	**In support (positive)**		
		The content was found to be easy to understand	“They are OK; even the explanation is straight forward.” [FGD 5] “The English they use is simple, they don’t use bombastic...” [FGD 5] “It is self-explanatory and not complicated English.” [FGD 4] “The language is understandable” [FGD 2]
	**Against (negative)**		
		The repetitive nature of the adherence to SMS text messages was found boring	“They sent the very same thing over and over so...no man!” [FGD 5] “Sometimes it was boring me, to be honest it was boring me. You do the same thing all the time.” [FGD 3]
	**Suggested adaptations**		
		To vary the phrasing of the adherence message from day to day (although some repetitiveness is inherent to the nature of these messages)	—^c^
**Length**			
	**In support (positive)**		
		The length of the messages was acceptable	“The length is not that much, like it’s very convenient, it doesn’t even take much of your time whereby you like now you have to sit down and read.” [FGD 2] “I think the long information is better.” [FGD 2]
	**Against (negative)**		
		The information messages were too long	“What I disliked is that they were lengthy.” [FGD 5] “When you are tired from school and all the studying then you receive a long text...its draining.” [FGD 1] “Sometimes they send lengthier ones and maybe you are busy so I didn’t like those.” [FGD 5]
	**Suggested adaptations**		
		To shorten the information messages or split them into multiple messages	“They should keep it brief and straight forward.” [FGD 5] “They should try and limit it and make sure they stick to the relevant key points and not add anything else.” [FGD 5] “So instead of sending one long message, they could send two different messages, one immediately after the other.” [FGD 1]

^a^Main conclusion: The intervention was found to be acceptable, easy to understand, and delivered through an acceptable medium; however, opinions differed according to personal preference and needs on frequency, timing, and length of the messages.

^b^FGD: focus group discussion.

^c^Indicates a suggested adaptation formulated by the study team inferred from the presented evidence from the FGD but without direct supporting evidence from the participants.

#### Interaction and Usability

**Figure 3:**
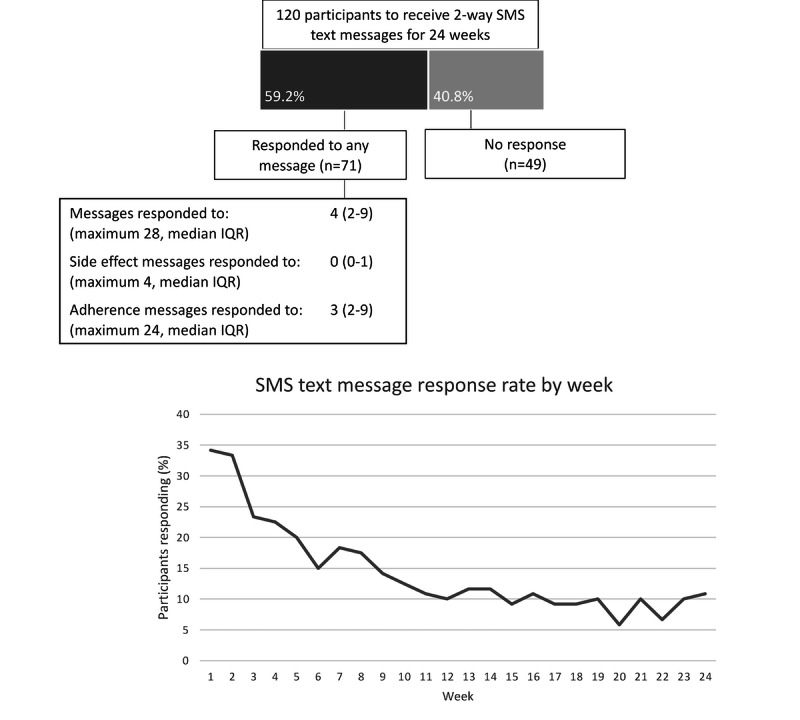
Use of 2-way SMS text messaging system by the number of SMS text message respondents and response rate by week.

On the basis of quantitative analysis of the data log of participants’ use of the 2-way SMS text messaging system, the participants’ interactions with the 2-way SMS text messaging intervention in terms of response rate were low, with only 59.2% (71/120) of participants replying to any 1 message (Figure 3). Use declined over the 6 months of the intervention, and 10.8% (13/120) of participants still receiving messages replied in week 24.

In the FGDs, participants described their main reasons for not using the reply function as being too busy, lack of interest, lack of airtime, not understanding whether they were required to reply, and a technical error (Table 4). Participants expressed that a fuller explanation of the reply function and the received weekly prepaid airtime was needed. Similarly, participants expressed that being too busy, a lack of interest, and technical issues were the main barriers to receiving and reading SMS text messages. In terms of technical issues, some participants received the message in 2 parts or only received half of the message, possibly because of the length of some of the health literacy messages.

**Table 4:** Qualitative evaluation of the usability of and interaction with the Healthy Life Trajectories Initiative Bukhali SMS text messaging intervention^a^.

Usability and interaction			Quotes
**Receiving and reading**			
	**In support of (positive)**		
		The SMS text message reached and was read by participants	“I always read them.” [FGD^b^ 1] “Yeah every time I got the message I read everything that is written on it.” [FGD 2] “If I saw that it is a different message I’d read the message.” [FGD 2]
	**Against (negative)**		
		Reasons for not reading the SMS text messages included being too busy, loss of interest, and technical issues in receiving the SMS text messages	“I won’t lie, when I am in class I just look at it and just put it back in my pocket.” [FGD 5] “I read them in the first week. I had the energy to; I would even sit down for it. But after that, no.” [FGD 5] “The phone I used to receive the SMS’s is broken, so...I no longer received the SMS’s” [FGD 1]
	**Suggested adaptations**		
		To send SMS text messages at a more convenient time (eg, earlier in the morning)	—^c^
		To resolve technical problems and unearthing and solving individual participants’ technical problems by asking for regular feedback about this	—
**Responding**			
	**In support of (positive)**		
		The respond function was used by some participants	“Even if I don’t have airtime I make a plan and respond because I know if I don’t respond and I have taken them it won’t be OK.” [FGD 4]
	**Against (negative)**		
		There was limited uptake for replying to the SMS text messages	“I only replied twice; all the other times I would just use the airtime for my own good.” [FGD 5] “I don’t look to check if I can reply or something. I don’t reply anyway.” [FGD 3]
		The main reasons for not replying were being busy, lack of interest, lack of airtime^d^, a technical error, and not understanding whether it is necessary to reply	“What prevented me from replying was that I would receive the SMS while I am in class and I can’t reply and by the time I get home; I have lost interest in the SMS.” [FGD 5] “I didn’t have time. And sometimes it was boring me.” [FGD 3] “Sometimes the airtime disappears to your airtime advances payments before you can reply” [FGD 5] “I used to respond but I can’t anymore, it just says error.” [FGD 1] “That is where I struggle to understand if they really want us to reply or you just leave it like that?” [FGD 1]
	**Suggested adaptations**		
		To justify and explain the reply function at the start of the intervention	“I think that you should explain...what should we reply? We must understand, you must give us the explanation.” [FGD 3]
		To improve participant knowledge and understanding of the reply function and the airtime received at the start of the intervention, possibly through a short training session	—
		To encourage participants to reply later even if they are busy at the moment of receiving the SMS text messages	—
		To evaluate the usefulness of the reply function in this setting	—

^a^Main conclusion: Although participants reported reading the SMS text messages, technical issues, a lack of time, and missing information were barriers to intervention usability, and participants expressed their use of the reply function was limited.

^b^FGD: focus group discussion.

^c^Indicates a suggested adaptation formulated by the study team inferred from the presented evidence from the FGD but without direct supporting evidence from the participants.

^d^Phone credit per minute.

#### Perceived Benefits Versus Unintended Consequences

Participants described experiencing benefits from the intervention, including feeling supported by the study team, being reminded to take the micronutrient supplements, and learning new concepts from the health literacy messages (Table 5). Unintended consequences associated with the health literacy messages that were described included feelings of fear about the information in the side effect messages and feeling patronized (made to feel “stupid”) by the messages. This may be because the SMS text messages reinforce content from the HeLTI *Bukhali* resources, and for participants with limited health literacy, this may feel that it highlights their lack of knowledge and understanding.

In general, participants did not feel the need to respond to the messages, although positive feelings about communicating through the reply function were expressed by one participant. In addition, some participants expressed feeling guilty for not responding to the messages or feeling like they might have been judged if they replied that they had not taken their supplements.

**Table 5:** Qualitative evaluation of the perceived benefits and consequences of the HeLTI^a^ Bukhali SMS text messaging intervention^b^.

Perceived benefits vs consequences			Quotes
**Overall intervention**			
	**In support of (positive)**		
		The intervention helped participants feel supported by the study team	“They make me feel like they care.” [FGD^c^ 4] “I liked it because it showed that you do follow up, you are not just giving us the supplements only.” [FGD 5] “They show that they want to be a part of this so that they can help us so that we can be focused and remember” [FGD 3]
	**Suggested adaptations**		
		To expand the SMS text messaging service to facilitate additional communication to address questions, monitoring of side effects, and delivery of the supplements	“If they can create a WhatsApp number where we can text them.” [FGD 5] “They must send me an SMS that by this date, we are going to be able to bring you your supplements.” [FGD 3] “To monitor [side effects] and...where it has boosted you so far” [FGD 3]
**Receiving health literacy messages**			
	**In support of (positive)**		
		The health literacy messages were found to be educational and filled a knowledge gap	“I am happy about it. It teaches me how to balance my diet, supplements and things like that” [FGD 1] “the SMS’s tell you exactly what they are for and what they do and help with” [FGD 4] “I actually got to learn the names of vitamins...for me it was exciting” [FGD 4] “At clinics or wherever they don’t give us that much information. Now you know that even the small things that you can be able to plant yourself you can be able to get iron and be sharp.” [FGD 3] “I think twice a week it’s perfect.” [FGD 2]
	**Against (negative)**		
		An unintended consequence of the health literacy messages was feelings of fear about side effects	“I think they should cut out the side effects one because sometimes it’s scarier to know that the medication you are about to take, you might come across this situation and that situation...might stop people from taking the supplements” [FGD 1]
		An overlap between the health literacy messages and HeLTI intervention resources was disadvantageous	“Because we already have the pamphlets; you make us feel like we are stupid by repeating the same thing; so I don’t like reading the same thing repetitively.” [FGD 5]
	**Suggested adaptations**		
		To review the phrasing of side effect messages for fear-inducing language and adapt where necessary	—^d^
		To ensure information messages complement and refer to HeLTI intervention resources	—
		To additionally disseminate the health literacy messages through platforms such as social media, television, and word of mouth	“Community health workers...with people, if someone knocks, you open for them and give them your attention, and then they educate.” [FGD 1] “Everything that there is on Facebook, I look at it.” [FGD 1]
**Receiving adherence messages**			
	**In support of (positive)**		
		Participants expressed that the adherence messages were an effective reminder	“They were helpful because sometimes you forget to take them but when you receive the message you remember.” [FGD 5] “We don’t receive them anymore and it shows because I forget to take them, but yes, they were a reminder.” [FGD 4] “And also, our lives are busy, a lot so the SMS sometimes reminds you that you have to wake up, do like this and like this and like this.” [FGD 3]
	**Against (negative)**		
		The repetitive nature of the adherence message was not found useful	“The information one I read, but the other one I don’t see it as necessary...because it’s the same thing.” [FGD 3]
	**Suggested adaptations**		
		(Please see above for suggestions for improved acceptability for adherence messages)	(Please see above for suggestions for improved acceptability for adherence messages)
**Responding to adherence messages**			
	**In support of (positive)**		
		Being able to reply to the messages felt beneficial for some	“I also feel good letting them know that I am taking the supplements.” [FGD 4]
	**Against (negative)**		
		Other participants did not perceive a benefit to responding to the messages	“Why should I have to reply?” [FGD 3] “I felt no type of way.” [FGD 3]
		An unintended consequence of the response option was feelings of guilt and judgment for not responding or taking supplements	“Because of that part that you cannot answer, you seem like a person who is not cooperative, but you do want to cooperate it’s just that you don’t have what they require [airtime].” [FGD 4] “I felt like they would judge me and say I’d ruin my body...When I didn’t take the supplements I felt a bit odd because I did not want to disappoint them.” [FGD 4]
	**Suggested adaptations**		
		To justify and explain the reply function at the start of the intervention	“I think that you should explain...what should we reply? We must understand, you must give us the explanation.” [FGD 3]
		To assess the need for a reply function in this intervention	—
		To thoroughly explain the nonjudgmental nature of the reply function, which is intended to help the participant.	—

^a^HeLTI: Healthy Life Trajectories Initiative.

^b^Main conclusion: Participants perceived practical, supportive, and educational benefits to receiving SMS text messages; however, there was little perceived benefit for the response option, and feelings of worry and fear were unintended consequences associated with the intervention.

^c^FGD: focus group discussion.

^d^Indicates a suggested adaptation formulated by the study team inferred from the presented evidence from the FGD but without direct supporting evidence from the participants.

#### Fidelity and Cost of Intervention Delivery

On the basis of the quantitative data log of received messages, airtime, and technical errors, there was only one participant to whom the SMS text messages were not delivered as intended because of a technical error, which was resolved at week 16 of the 24 weeks after updating the participant’s number and provider. As indicated in Table 4, in the FGDs, participants sometimes reported receiving messages in 2 parts or only receiving half a message, particularly when using nonsmartphone models. Some also reported having technical problems when trying to reply to the message. However, the exact cause or number of cases in which this occurred could not be determined.

In terms of receiving weekly prepaid airtime, a few participants experienced technical problems, with 4.2% (5/120) of participants experiencing repeated technical errors. Of these, 3 were because of the initial recording and use of the wrong cell network by the research team. One was because of a change in the phone number. For these 4 participants, errors were resolved within 2 months of onset. For the fifth participant with a repeated technical error, technical failure could not be resolved, likely because they had a nonchargeable SIM card.

On the basis of the cost log for the intervention, delivering the SMS text messaging intervention cost ZAR 71.65 (US $4.65) per participant or, on average, ZAR 245.64 (US $15.93) per week for the 35 weeks that SMS text messages were sent. When including the weekly prepaid airtime, the intervention cost per participant was ZAR 266.26 (US $17.27) or ZAR 912.91 (US $59.21) per week.

#### Indicative Effect of the Bukhali Text Messaging Intervention

**Figure 4:**
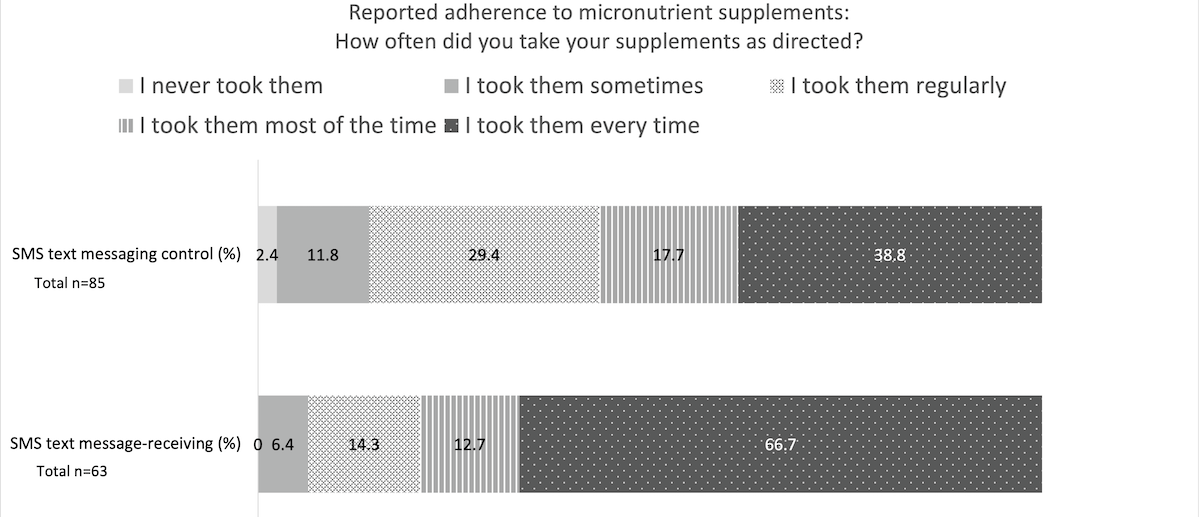
Bar graph comparing the SMS text message–receiving and SMS text messaging control group participants’ self-reported adherence at follow-up.

**Figure 5:**
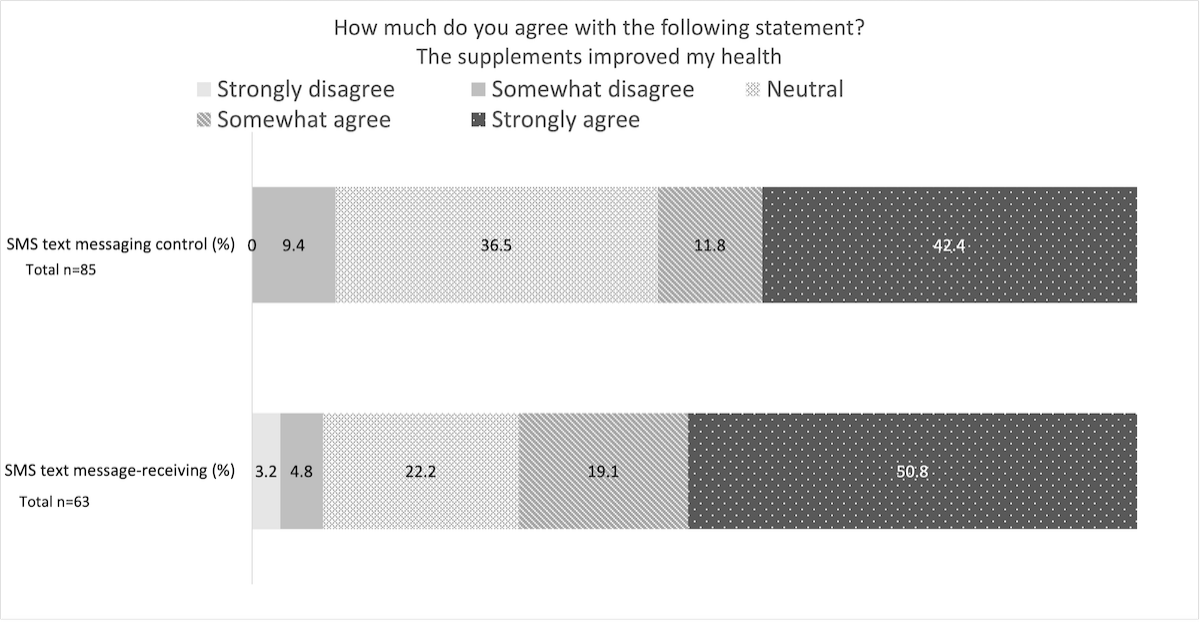
Bar graph comparing the SMS text message–receiving and SMS text messaging control group’s attitudes toward supplements improving their health at follow-up.

This section reports the results of preliminary, nonrandomized comparisons between the SMS text message–receiving and SMS text messaging control groups. As shown in Figure 4, at follow-up, self-reported adherence was higher in the SMS text message–receiving group (42/63, 67% reported taking supplements every time) than in the SMS text messaging control group (33/85, 39%; *P*=.02). In addition, a larger percentage of the SMS text message–receiving group (32/63, 51%) strongly agreed that the supplements had a positive impact on their health than the SMS text messaging control group (36/85, 42%; *P*=.09; Figure 5). The main reasons for sometimes or often missing supplements in both groups were traveling (and therefore not having access to the supplement) and forgetting; however, fewer participants in the SMS text message–receiving group reported missing their supplements for these reasons. Moreover, a lower number of participants in the SMS text message–receiving group reported having fears about taking the supplements than in the SMS text messaging control group (4/63, 6% vs 16/77, 21%; *P*=.02), although the number of participants reporting a side effect in the SMS text message–receiving group was nonstatistically significantly higher than in the SMS text messaging control group (13/64, 20% vs 9/84, 11%,). In addition, statistically significantly more participants in the SMS text message–receiving group agreed or strongly agreed that the micronutrient supplements were explained to them well (59/64, 92% vs 66/87, 76% in the SMS text messaging control group; *P*=.03).

The mean difference in hemoglobin from baseline to follow-up (Δhemoglobin) was negative in the SMS text messaging control group (mean −0.46, SD 1.59), whereas in the SMS text message–receiving group, Δhemoglobin was positive (mean 0.52, SD 2.18; *P*=.002). This difference in hemoglobin levels between the 2 groups remained statistically significant when the 2 participants with severe anemia at baseline were excluded from the SMS text message–receiving group (*P*=.003).

Exploring this finding further with a mixed linear model, we found that the altitude-adjusted hemoglobin increased more between baseline and follow-up in the SMS text message–receiving group than in the SMS text messaging control group, as indicated by the interaction term in the model (1.03, 95% CI 0.49-1.57; *P*<.001; Table 6), although absolute the hemoglobin level was lower in the SMS text message–receiving group when adjusted for time (−0.70, 95% CI −1.13 to −0.28; *P*=.001). These results remained statistically significant and were not attenuated after correcting for differences present at baseline (employment status and level of education; Table S2, Multimedia Appendix 1).

**Table 6:** Linear mixed modeling for altitude-adjusted hemoglobin, adjusting for intervention exposure, time (baseline vs follow-up), and the interaction between time and intervention exposure^a,b^.

Model	Coefficient (95% CI)	*P* value
SMS text message–receiving group	−0.70 (−1.13 to −0.28)	.001
Time (baseline to follow-up)	−0.51 (−0.88 to −0.14)	.007
SMS text message–receiving group×time	1.03 (0.49 to 1.57)	<.001

^a^No other covariables other than those shown were included in the model. SMS text message–receiving group×time indicates the interaction term between the SMS text messaging intervention exposure and time of measurement (baseline vs follow-up).

^b^Average observations per group 1.7; *P* value model=.001.

### The Phila Impilo Yakho Kangcono Radio Serial Intervention

#### Objective 2: Acceptability of the Radio Serial and Facebook Page

Participants expressed that they found the content of the messages educational and relatable and that they enjoyed the narrative aspect of the radio serial as a way of communicating health messages (Table 7). The participants reported not having previously heard the radio serial on the national radio station (Ukhozi FM) or on the local station (Jozi FM).

**Table 7:** Qualitative evaluation of the acceptability of the Phila Impilo Yakho Kangcono radio serial^a^.

Acceptability radio serial			Quotes
**Delivery**			
	**In support (positive)**		
		Participants liked the delivery of health messages through a narrative radio serial	“On radio you get to hear people’s voices and emotions and by hearing what they’re saying maybe you can change your diet, your ways.” [FGD^b^ 2]
		Participants liked the associated Facebook page	“It’s informative, it’s youthful.” [FGD 4] “I find it really inspirational because we learnt about things we did not know about.” [FGD 5]
		Participants reported listening to the radio occasionally and in specific situations	“You find that people don’t know how you get diabetes,...so it’s educational.” [FGD 2] “I personally think it [relationship between characters in serial] is realistic.” [FGD 1] “she [character] is the one who educates others...She also wants to learn so that she helps her mother, she’s so supportive.” [FGD 1]
	**Against (negative)**		
		Participants reported not having previously heard the radio serial	“I listen to radio but have never heard it before.” [FGD 3] “I don’t listen to radio.” [FGD 1]
	**Suggested adaptations**		
		To increase awareness of the radio serial	“And word of mouth will help, just to say there’s this show on the radio.” [FGD 2]
**Content**			
	**In support of (positive)**		
		The content and characters of the radio serial were found to be relatable and acceptable	“You find that people don’t know how you get diabetes,...so it’s educational.” [FGD 2] “I personally think it [relationship between characters in serial] is realistic.” [FGD 1] “she [character] is the one who educates others...She also wants to learn so that she helps her mother, she’s so supportive.” [FGD 1]
	**Suggested adaptations**		
		To incorporate additional health literacy topics of interest, including exercise, hygiene, sexually transmitted illnesses, cancer, COVID-19, gender-based violence, and mental health	“How to deal with your anxiety. Because with me when I walk the streets, I get scared...I overthink a lot especially with my schoolwork” [FGD 3] “I think cancer as well because we think it’s only for older people and we also get cancer now.” [FGD 5] “COVID-19 as well” [FGD 5]

^a^Main conclusion: Participants found the delivery radio serial and associated Facebook page acceptable and the content likable and relatable.

^b^FGD: focus group discussion.

#### Advantages and Disadvantages of SMS Text Messaging, Radio, and Facebook Page

An overview of the advantages and disadvantages of the different health messaging media based on the FGD data is provided in Table 8. Although participants expressed concerns about the accessibility of data-requiring messaging platforms such as Facebook and had not visited the Facebook page associated with the radio serial before the FGD, participants liked that it provided a community with whom they could interact.

**Table 8:** Qualitative results comparing the advantages and disadvantages of health messaging in the social messaging media, including SMS text messaging, radio, and a Facebook page.

Media				Quotes
**Advantages**				
	**SMS text messaging**			
		Participants frequently use their phones	“The fact that I’m always on my phone and it’s easy to access” [FGD^a^ 4] “I always have time [to read the message] because I am always on my phone” [FGD 1]	
		Does not require access to data	“What if you don’t have data for social media? SMS doesn’t need data.” [FGD 1]	
	**Radio**			
		Ability to listen to health messages through a story	“On radio you get to hear people’s voices and emotions” [FGD 2] “I think radio communicates better than the SMS’s because sometimes you are lazy to read SMS’s but listening is better.” [FGD 5]	
		Does not require access to data and can be accessed anywhere	“On radio it’s an advantage...you can just switch on your radio, headsets and listen, whether you’re in a taxi, whether you’re doing something and you listen” [FGD 2]	
	**Facebook page**			
		The community associated with the page	“Some people go through a lot...it can actually help people open up about themselves.” [FGD 3] “I think with this page we can, people can talk and express their feelings.” [FGD 2]	
		Ability to find past content (eg, past radio serial episodes)	“[I prefer] social media. When you miss this show, nowadays there’s load shedding then when you miss the show they must repeat it so that you can listen to it again.” [FGD 2]	
**Disadvantages**				
	**SMS text messaging**			
		Some participants indicated a preference for phone calls	“I prefer that they call; these older models are very problematic...so I prefer them to call, the same way they call when they run sessions.” [FGD 5]	
	**Radio**			
		Not all participants listen to the radio frequently	“We don’t often listen to the radio so I prefer the SMS” [FGD 4]	
		Inability to find past content	“Nowadays there’s load shedding [power cuts] then when you miss the show they must repeat it so that you can listen to it again.” [FGD 2]	
	**Facebook page**			
		Data requiring	“Social media, we always don’t have data.” [FGD 2] “Not all of us can access the internet.” [FGD 4]	
		Participants need to proactively log in	“When you get the notification you take your phone and check, but with Facebook, a week might go by without you logging in.” [FGD 1] “I don’t have Facebook I just deleted it so I prefer to listen to radio.” [FGD 2]	

^a^FGD: focus group discussion.

## Discussion

### Principal Findings and Implications

In this nested feasibility study, the HeLTI *Bukhali* SMS text messaging intervention, aimed at improving preconception adherence to micronutrient supplements, was found to be acceptable, beneficial, and usable by participants. Indicative effect results suggest a promising potential effect of the SMS text messaging intervention on self-reported supplement adherence and changes in hemoglobin from baseline to follow-up, although these are not based on a randomized comparison. These findings are in line with evidence from high-income countries on the acceptability and potential of SMS text messaging interventions to support behavior change in various life stages and contribute to the existing evidence, although less conclusive, for SMS text messaging interventions from low- and middle-income countries [20,21,27,28]. Finally, the health-promoting radio serial was found to be acceptable for young women.

Although participants reported finding the SMS text messages easy to understand and helpful, we received several suggestions for adapting the SMS text messaging intervention. This included a preference for receiving messages in the early morning on days they were meant to take their supplement. Although personalizing the timing and frequency of SMS text message delivery could negatively affect the scalability of the intervention [47], it may be feasible to send more frequent messages to those participants taking supplements daily, as suggested in the FGDs. As the SMS text messaging intervention was automated, it required few resources and was not demanding on implementation staff, increasing its practicality in limited-resource settings such as ours. However, introducing a nonautomated aspect to the intervention, for example, through a *help desk*, which participants could reach by replying to a number, could help manage the technical challenges experienced by participants and facilitate communication with the study team. Existing evidence also suggests that a nonautomated, personalized element may increase interaction with and the effect size of the intervention, although this remains to be tested from a cost-effectiveness perspective [48]. It is important to establish the feasibility and affordability of such a service in low- and middle-income settings, given that automated systems could be more scalable and sustainable in resource-constrained environments. Furthermore, in the context of the HeLTI *Bukhali* trial, there are likely other intervention priorities that would take precedence over such a service, particularly in terms of the allocation of human resources. However, there could be less resource-intensive ways of integrating these learnings into the intervention implementation, such as reminding those delivering the intervention to follow up more intentionally about the delivery of SMS text messages with participants.

In our study, the 2-way reply function of the SMS text messaging intervention was underused, with only 10.8% (13/120) of participants using the reply function in the final week of the intervention. A study evaluating a 2-way SMS text messaging intervention for weight loss in postpartum women in a high-income setting found good engagement with a comparable *yes or no* SMS text message reply option, with most participants replying to over half of the messages and high engagement contributing to the outcome of interest [28]. In a low- to middle-income setting, some evidence suggests that a 2-way SMS text messaging intervention could be effective if engagement is high enough [49]; however, a study of a 2-way SMS text messaging intervention for improved HIV care in pregnant and postpartum women in Kenya found a similarly low response frequency [50]. Qualitative research from Uganda indicated similar barriers to responding to a 2-way SMS text messaging intervention, as expressed by our participants, including a lack of airtime, being too busy, and phone-specific issues [51]. Whether these barriers can be minimized through more extensive instructions, explanations, participant encouragement at intervention initiation, or the use of alternative media such as unstructured supplementary service data would require further assessment before this aspect of the intervention can be recommended in our setting. Despite the low response rate, the SMS text messaging intervention was found to be acceptable and showed a potentially indicative effect. Therefore, a 2-way function may not be an essential component of an intervention aimed at improving supplement intake, as opposed to more complex behavior change targets.

In terms of population-level health promotion messaging, our participants indicated a preference for the narrative and accessible nature of the *Phila Impilo Yakho Kangcono* radio serial. Radio health messaging has been found to increase health knowledge and awareness in the context of, among others, maternal health, HIV prevention research, and child health in low- and middle-income settings, including Southern Africa [52-56]. However, we found that not all participants listened to the radio frequently, indicating the need for research on the reach of such a radio-based health promotion campaign among young women in our setting. By combining media, directed health messaging (such as SMS text messaging) could be used to increase young women’s awareness of new radio and social media campaigns to improve their reach and effectiveness [57].

### Strengths and Limitations

To the best of our knowledge, this is the first study in our setting to evaluate social messaging through individual-level SMS text messaging intervention and population-level radio serial for preconception health promotion. However, a limitation of this study is that more in-depth data on the acceptability, feasibility, and impact of the radio serial were not available as participants’ exposure was limited to 1 or 2 episodes, which they heard for the first time during the FGDs. Another limitation is the high rate of loss to follow-up and the presence of missing data, which threatens the validity of the findings regarding the indicative effect of the SMS text messaging intervention. Moreover, the loss to follow-up may indicate that collecting data on these end points might be challenging in the context of a full randomized controlled trial. Although a portion of the loss to follow-up can likely be explained by the unique circumstances of the COVID-19 pandemic, additional methods to limit withdrawal, increase participant motivation for participation, and prevent loss of contact (eg, through community engagement) could be useful to explore. The withdrawal and loss-to-follow-up rates were higher in the SMS text message–receiving group than in the SMS text messaging control group, which could be because of the greater impact of COVID-19 later in the pandemic (with data collected in 2021 vs 2020). Although there was no indication from the participants that they were withdrawing in response to the SMS text messaging intervention, further (randomized) analysis could help confirm this. The evaluation of the indicative effect of the SMS text messaging intervention was not conducted between the 2 randomized groups, and the baseline difference in hemoglobin levels between the SMS text message–receiving and SMS text messaging control groups, although adjusted for in the linear mixed model, may have affected our results. Therefore, a fully randomized controlled exploration of the effectiveness, as well as the cost-effectiveness, of the intervention is still needed.

The FGDs were moderated by trained research staff from Soweto who were not involved in the development of the intervention. The researcher performing the initial coding and qualitative analysis (LMS) was a cultural outsider as a White European woman, which may have affected nuanced aspects of data interpretation, although the outcomes were reviewed by other members of the research team [58]. LMS was also involved in the study implementation and management (although not the development of either intervention), which may have contributed to observer bias [59]. Nevertheless, this study contributes to the understanding of women’s perceptions of preconception health messaging strategies in our setting.

### Conclusions

In this feasibility study, young women found that an SMS text messaging intervention aimed at increasing adherence to preconception micronutrient supplements was acceptable. In addition, mass media health promotion through a radio serial aimed at young people was found to be acceptable and relatable. Participants found the HeLTI *Bukhali* SMS text messaging intervention beneficial and useful. The intervention may also help improve self-reported adherence, attitudes toward supplements, and measured hemoglobin levels at follow-up, although these results are based on a limited, nonrandomized comparison. Although these findings suggest a previously unexplored potential for SMS text messaging interventions to support improvements in preconception micronutrient supplement adherence, further studies in the form of randomized trials are still needed. In addition, some refinements based on participant suggestions and reassessment of the 2-way SMS text messaging component are needed. Social messaging interventions for improving the health and nutrition of young women could consider how directed messaging (such as SMS text messaging) and mass messaging strategies (such as radio) may be used to complement each other to promote the health of young women in the preconception period.

## Appendix

Multimedia Appendix 1 The main baseline characteristics in the followed-up versus lost to follow-up group and linear mixed modeling for altitude-adjusted hemoglobin, adjusting for intervention exposure, time (baseline vs follow-up), the interaction between time and intervention exposure, and employment status and level of education.

## Abbreviations

FGD: focus group discussion
HAPA: Health Actions Process Approach
HeLTI: Healthy Life Trajectories Initiative
REDCap: Research Electronic Data Capture
TIDieR: Template for Intervention Description and Replication

## References

[1] Stephenson J, Heslehurst N, Hall J, Schoenaker DA, Hutchinson J, Cade JE, Poston L, Barrett G, Crozier SR, Barker M, Kumaran K, Yajnik CS, Baird J, Mishra GD (2018). Before the beginning: nutrition and lifestyle in the preconception period and its importance for future health. Lancet.

[2] Caut C, Leach M, Steel A (2020). Dietary guideline adherence during preconception and pregnancy: a systematic review. Matern Child Nutr.

[3] Black RE, Victora CG, Walker SP, Bhutta ZA, Christian P, de Onis M, Ezzati M, Grantham-McGregor S, Katz J, Martorell R, Uauy R (2013). Maternal and child undernutrition and overweight in low-income and middle-income countries. Lancet.

[4] Toivonen KI, Lacroix E, Flynn M, Ronksley PE, Oinonen KA, Metcalfe A, Campbell TS (2018). Folic acid supplementation during the preconception period: a systematic review and meta-analysis. Prev Med.

[5] Liu D, Cheng Y, Dang S, Wang D, Zhao Y, Li C, Li S, Lei F, Qu P, Mi B, Zhang R, Li J, Zeng L, Yan H (2019). Maternal adherence to micronutrient supplementation before and during pregnancy in Northwest China: a large-scale population-based cross-sectional survey. BMJ Open.

[6] Masho SW, Bassyouni A, Cha S (2016). Pre-pregnancy obesity and non-adherence to multivitamin use: findings from the National Pregnancy Risk Assessment Monitoring System (2009-2011). BMC Pregnancy Childbirth.

[7] Malek L, Umberger W, Makrides M, Zhou SJ (2016). Poor adherence to folic acid and iodine supplement recommendations in preconception and pregnancy: a cross-sectional analysis. Aust N Z J Public Health.

[8] (2013). South African National Health and Nutrition Examination Survey (SANHANES-1).

[9] Prioreschi A, Wrottesley S, Draper CE, Tomaz SA, Cook CJ, Watson ED, Van Poppel MN, Said-Mohamed R, Norris SA, Lambert EV, Micklesfield LK (2017). Maternal and early life nutrition and physical activity: setting the research and intervention agenda for addressing the double burden of malnutrition in South African children. Glob Health Action.

[10] Bosire E, Ware L, Draper C, Amato B, Kapueja L, Lye S, Norris S (2021). Young women’s perceptions of life in urban south Africa: contextualising the preconception knowledge gap. Afr J Reprod Health.

[11] Broadcast Research Council of South Africa (2020). BRC RAM™ Release Presentation April '19-March '20.

[12] Saaka M, Wemah K, Kizito F, Hoeschle-Zeledon I (2021). Effect of nutrition behaviour change communication delivered through radio on mothers' nutritional knowledge, child feeding practices and growth. J Nutr Sci.

[13] Schmid KL, Rivers SE, Latimer AE, Salovey P (2008). Targeting or tailoring? Maximizing resources to create effective health communications. Mark Health Serv.

[14] The state of the ICT sector in South Africa, 2019. Independent Communications Authority of South Africa.

[15] Leon N, Surender R, Bobrow K, Muller J, Farmer A (2015). Improving treatment adherence for blood pressure lowering via mobile phone SMS-messages in South Africa: a qualitative evaluation of the SMS-text Adherence SuppoRt (StAR) trial. BMC Fam Pract.

[16] Zhuang Q, Chen F, Wang T (2020). Effectiveness of short message service intervention to improve glycated hemoglobin control and medication adherence in type-2 diabetes: a meta-analysis of prospective studies. Prim Care Diabetes.

[17] Thakkar J, Kurup R, Laba T, Santo K, Thiagalingam A, Rodgers A, Woodward M, Redfern J, Chow CK (2016). Mobile telephone text messaging for medication adherence in chronic disease: a meta-analysis. JAMA Intern Med.

[18] Boksmati N, Butler-Henderson K, Anderson K, Sahama T (2016). The effectiveness of SMS reminders on appointment attendance: a meta-analysis. J Med Syst.

[19] Spohr SA, Nandy R, Gandhiraj D, Vemulapalli A, Anne S, Walters ST (2015). Efficacy of SMS text message interventions for smoking cessation: a meta-analysis. J Subst Abuse Treat.

[20] Demena BA, Artavia-Mora L, Ouedraogo D, Thiombiano BA, Wagner N (2020). A systematic review of mobile phone interventions (SMS/IVR/calls) to improve adherence and retention to antiretroviral treatment in low-and middle-income countries. AIDS Patient Care STDS.

[21] Linde DS, Korsholm M, Katanga J, Rasch V, Lundh A, Andersen MS (2019). One-way SMS and healthcare outcomes in Africa: systematic review of randomised trials with meta-analysis. PLoS One.

[22] Leon N, Namadingo H, Cooper S, Bobrow K, Mwantisi C, Nyasulu M, Sicwebu N, Crampin A, Levitt N, Farmer A (2021). Process evaluation of a brief messaging intervention to improve diabetes treatment adherence in sub-Saharan Africa. BMC Public Health.

[23] Steward WT, Agnew E, de Kadt J, Ratlhagana MJ, Sumitani J, Gilmore HJ, Grignon J, Shade SB, Tumbo J, Barnhart S, Lippman SA (2021). Impact of SMS and peer navigation on retention in HIV care among adults in South Africa: results of a three-arm cluster randomized controlled trial. J Int AIDS Soc.

[24] Govender K, Beckett S, Masebo W, Braga C, Zambezi P, Manhique M, George G, Durevall D (2019). Effects of a short message service (SMS) intervention on reduction of HIV risk behaviours and improving HIV testing rates among populations located near roadside wellness clinics: a cluster randomised controlled trial in South Africa, Zimbabwe and Mozambique. AIDS Behav.

[25] Mukund Bahadur KC, Murray PJ (2010). Cell phone short messaging service (SMS) for HIV/AIDS in South Africa: a literature review. Stud Health Technol Inform.

[26] Skinner D, Delobelle P, Pappin M, Pieterse D, Esterhuizen TM, Barron P, Dudley L (2018). User assessments and the use of information from MomConnect, a mobile phone text-based information service, by pregnant women and new mothers in South Africa. BMJ Glob Health.

[27] Barron P, Peter J, LeFevre AE, Sebidi J, Bekker M, Allen R, Parsons AN, Benjamin P, Pillay Y (2018). Mobile health messaging service and helpdesk for South African mothers (MomConnect): history, successes and challenges. BMJ Glob Health.

[28] McGirr C, Rooney C, Gallagher D, Dombrowski SU, Anderson AS, Cardwell CR, Free C, Hoddinott P, Holmes VA, McIntosh E, Somers C, Woodside JV, Young IS, Kee F, McKinley MC (2020). Text messaging to help women with overweight or obesity lose weight after childbirth: the intervention adaptation and SMS feasibility RCT. Public Health Res.

[29] van Dijk MR, Koster MP, Oostingh EC, Willemsen SP, Steegers EA, Steegers-Theunissen RP (2020). A mobile app lifestyle intervention to improve healthy nutrition in women before and during early pregnancy: single-center randomized controlled trial. J Med Internet Res.

[30] Norris SA, Draper CE, Prioreschi A, Smuts CM, Ware LJ, Dennis C, Awadalla P, Bassani D, Bhutta Z, Briollais L, Cameron DW, Chirwa T, Fallon B, Gray CM, Hamilton J, Jamison J, Jaspan H, Jenkins J, Kahn K, Kengne AP, Lambert EV, Levitt N, Martin M-C, Ramsay M, Roth D, Scherer S, Sellen D, Slemming W, Sloboda D, Szyf M, Tollman S, Tomlinson M, Tough S, Matthews SG, Richter L, Lye S (2022). Building knowledge, optimising physical and mental health and setting up healthier life trajectories in South African women (Bukhali): a preconception randomised control trial part of the Healthy Life Trajectories Initiative (HeLTI). BMJ Open.

[31] Draper C, Prioreschi A, Ware L, Lye S, Norris S (2020). Pilot implementation of ‘Bukhali’: a preconception health trial in South Africa. SAGE Open Med.

[32] Black C, Lawrence W, Cradock S, Ntani G, Tinati T, Jarman M, Begum R, Inskip H, Cooper C, Barker M, Baird J (2014). Healthy conversation skills: increasing competence and confidence in front-line staff. Public Health Nutr.

[33] Schwarzer R (2008). Modeling health behavior change: how to predict and modify the adoption and maintenance of health behaviors. Applied Psychol.

[34] Crombie IK, Irvine L, Williams B, Sniehotta FF, Petrie D, Jones C, Norrie J, Evans JM, Emslie C, Rice PM, Slane PW, Humphris G, Ricketts IW, Melson AJ, Donnan PT, Hapca SM, McKenzie A, Achison M (2018). Texting to Reduce Alcohol Misuse (TRAM): main findings from a randomized controlled trial of a text message intervention to reduce binge drinking among disadvantaged men. Addiction.

[35] Asgari S, Abbasi M, Hamilton K, Chen Y, Griffiths MD, Lin C, Pakpour AH (2021). A theory-based intervention to promote medication adherence in patients with rheumatoid arthritis: a randomized controlled trial. Clin Rheumatol.

[36] Live your best life. Facebook.

[37] Cresswell JW, Cresswell JD (2018). Research Design: Qualitative, Quantitative, and Mixed Methods Approaches.

[38] Moore GF, Audrey S, Barker M, Bond L, Bonell C, Hardeman W, Moore L, O'Cathain A, Tinati T, Wight D, Baird J (2015). Process evaluation of complex interventions: medical research council guidance. BMJ.

[39] Peters DH, Adam T, Alonge O, Agyepong IA, Tran N (2013). Implementation research: what it is and how to do it. BMJ.

[40] Harris PA, Taylor R, Thielke R, Payne J, Gonzalez N, Conde JG (2009). Research electronic data capture (REDCap)--a metadata-driven methodology and workflow process for providing translational research informatics support. J Biomed Inform.

[41] Wehler C, Scott R, Anderson J (1992). The community childhood hunger identification project: a model of domestic hunger—demonstration project in Seattle, Washington. J Nutrition Educ.

[42] Kehoe S, Wrottesley SV, Ware L, Prioreschi A, Draper C, Ward K, Lye S, Norris S (2021). Food insecurity, diet quality and body composition: data from the Healthy Life Trajectories Initiative (HeLTI) pilot survey in urban Soweto, South Africa. Public Health Nutr.

[43] Haemoglobin concentrations for the diagnosis of anaemia and assessment of severity. World Health Organization.

[44] Silubonde TM, Baumgartner J, Ware LJ, Malan L, Smuts CM, Norris S (2020). Adjusting haemoglobin values for altitude maximizes combined sensitivity and specificity to detect iron deficiency among women of reproductive age in Johannesburg, South Africa. Nutrients.

[45] Braun V, Clarke V (2006). Using thematic analysis in psychology. Qual Res Psychol.

[46] Gale NK, Heath G, Cameron E, Rashid S, Redwood S (2013). Using the framework method for the analysis of qualitative data in multi-disciplinary health research. BMC Med Res Methodol.

[47] Hall AK, Cole-Lewis H, Bernhardt JM (2015). Mobile text messaging for health: a systematic review of reviews. Annu Rev Public Health.

[48] Sahin C, Courtney KL, Naylor PJ, E Rhodes R (2019). Tailored mobile text messaging interventions targeting type 2 diabetes self-management: a systematic review and a meta-analysis. Digit Health.

[49] Harrington EK, Drake AL, Matemo D, Ronen K, Osoti AO, John-Stewart G, Kinuthia J, Unger JA (2019). An mHealth SMS intervention on postpartum contraceptive use among women and couples in Kenya: a randomized controlled trial. Am J Public Health.

[50] Pintye J, Rogers Z, Kinuthia J, Mugwanya KK, Abuna F, Lagat H, Sila J, Kemunto V, Baeten JM, John-Stewart G, Unger JA (2020). Two-way short message service (SMS) communication may increase pre-exposure prophylaxis continuation and adherence among pregnant and postpartum women in Kenya. Glob Health Sci Pract.

[51] Rana Y, Haberer J, Huang H, Kambugu A, Mukasa B, Thirumurthy H, Wabukala P, Wagner GJ, Linnemayr S (2015). Short message service (SMS)-based intervention to improve treatment adherence among HIV-positive youth in Uganda: focus group findings. PLoS One.

[52] Nyirenda D, Makawa TC, Chapita G, Mdalla C, Nkolokosa M, O'byrne T, Heyderman R, Desmond N (2018). Public engagement in Malawi through a health-talk radio programme ' Umoyo nkukambirana': a mixed-methods evaluation. Public Underst Sci.

[53] Radoff K, Levi A, Thompson L (2013). A radio-education intervention to improve maternal knowledge of obstetric danger signs. Rev Panam Salud Publica.

[54] Medeossi B, Stadler J, Delany-Moretlwe S (2014). 'I heard about this study on the radio': using community radio to strengthen Good Participatory Practice in HIV prevention trials. BMC Public Health.

[55] Achia TN (2015). Tobacco use and mass media utilization in sub-Saharan Africa. PLoS One.

[56] Monterrosa EC, Frongillo EA, González de Cossío T, Bonvecchio A, Villanueva MA, Thrasher JF, Rivera JA (2013). Scripted messages delivered by nurses and radio changed beliefs, attitudes, intentions, and behaviors regarding infant and young child feeding in Mexico. J Nutr.

[57] Rimer BK, Kreuter MW (2006). Advancing tailored health communication: a persuasion and message effects perspective. J Commun.

[58] Abimbola S (2019). The foreign gaze: authorship in academic global health. BMJ Glob Health.

[59] Mahtani K, Spencer EA, Brassey J, Heneghan C (2018). Catalogue of bias: observer bias. BMJ Evid Based Med.

