# Supplementary material for: Evaluation of a Text Messaging Intervention to Promote Preconception Micronutrient Supplement Use: Feasibility Study Nested in the Healthy Life Trajectories Initiative Study in South Africa
Source: JMIR Form Res. 2022 Aug 18;6(8):e37309. doi: 10.2196/37309 (PMC9437786; doi:10.2196/37309)
Supplement: Multimedia Appendix 1 [file formative_v6i8e37309_app1.docx]

**Multimedia Appendix 1.** The main baseline characteristics in the followed-up versus lost to follow-up group and linear mixed modeling for altitude-adjusted hemoglobin, adjusting for intervention exposure, time (baseline vs follow-up), the interaction between time and intervention exposure, and employment status and level of education.

**Supplementary Table S1:** Baseline characteristics of 1) participants with data at follow-up vs those lost to follow-up, and 2) participants with Hb data at follow-up vs those lost to Hb follow-up.

|  | n | Any data at follow-up | n | Lost to follow-up | n | Hb data at follow-up | n | Lost to Hb follow-up |
| --- | --- | --- | --- | --- | --- | --- | --- | --- |
|  |  |  |  |  |  |  |  |  |
| **Age at baseline**  **Hb altitude adjusted (g/dL)**  **BMI**  **Attitudes towards supplements at baseline**  1) How sure are you that you will be able to take all or most of your supplements as directed?  Not at all  Somewhat sure  Very/extremely sure  2) How sure are you that the supplements will have a positive effect on your health?  Not at all  Somewhat sure  Very/extremely sure  **Demographic characteristics**  Previous live births  0  1  ≥2  Born in SA  Unemployed (and not studying)  Graduated high school  Food security  At risk (1)  Food insecure ( ≥2)  Current frequent smoker  Smoked in past year | 185  185  132  185  185  185  185  185  185  185 | 21 (29-24)  12.4 (11.8-13.6)  23.5 (20.7-28.1)  0 (0)  7 (5.3)  125 (94.7)  2 (1.5)  19 (14.4)  111 (84.1)  105 (56.8)  55 (29.7)  25 (13.5)  185 (100)  143 (77.3)  118 (63.8)  42 (22.7)  68 (36.8)  24 (13.0)  139 (75.1) | 55  55  39  54  55  55  55  55  55  55 | 22 (19-24)  12.3 (11.6-13.1)  24.9 (22.4-29.0)  1 (2.6)  4 (10.3)  34 (87.2)  2 (5.1)  9 (23.1)  28 (71.8)  26 (48.2)  21 (38.9)  7 (13.0)  53 (96.4)*^1^  40 (72.7)  27 (49.1)  14 (25.5)  17 (30.9)  6 (10.9)  47 (85.5) | 168  168  119  168  168  168  168  168  168  168 | 21 (19-24)  12.4 (11.8-13.6)  23.3 (20.7-28.1)  0 (0)  6 (5.0)  113 (95.0)  2 (1.7)  19 (16.0)  98 (82.4)  97 (57.7)  48 (28.6)  23 (13.7)  168 (100)  129 (76.8)  103 (61.3)  37 (22.0)  62. (36.9)  22 (13.1)  43 (25.6) | 72  72  52  71  72  72  72  72  72  72 | 22 (20-24.5)  12.5 (11.7-13.5)  24.8 (20.9-29.1)  1 (1.9)  5 (9.6)  46 (88.5)  2 (3.9)  9 (17.3)  41 (78.9)  34 (47.9)  28 (39.4)  9 (12.7)  70 (97.2)*^2^  54 (75.0)  42 (58.3)  19 (26.4)  23 (31.9)  8 (11.1)  11 (15.3) |

Results provided as n(%) or median (IQR range). Statistically significant differences indicates as *^1^ between participants with any data at follow and those lost to follow-up; *^2^ between participants with Hb data at follow-up and those lost to Hb follow-up. Significance determined at *P*<0.05, using a Mann-Whitney U-test for continuous outcomes and chi^2^ statistic/Fisher’s exact test (if cell count <5) for categorical outcomes.

**Supplementary Table S2:** Linear mixed modelling for altitude adjusted Hb, adjusting for intervention exposure, time (baseline vs follow-up), the interaction between these variables, being unemployed at baseline, and having graduated from high school at baseline.

|  | **Coefficient (95% CI)** | ***P*-value** |
| --- | --- | --- |
|  |  |  |
| SMS-receiving | -0.70 (-1.13- -0.27) | 0.001 |
| Time (baseline to follow-up) | -0.51 (-0.88- - 0.14) | 0.007 |
| SMS-receiving#time | 1.03 (0.49- 1.57) | <0.001 |
| Unemployed | -0.10 (-0.55-0.34) | 0.641 |
| Graduated high school | -0.09 (-0.47-0.29) | 0.646 |
| Average observations per group: 1.7  *P*-value model: 0.004 | | |

*No other covariables not shown were included in the model. “SMS-receiving#time” indicates the interaction term between SMS-intervention exposure and time of measurement (baseline vs follow-up).*
